# *COBLL1* and *IRS1* Gene Polymorphisms and Placental Expression in Women with Gestational Diabetes

**DOI:** 10.3390/biomedicines10081933

**Published:** 2022-08-09

**Authors:** Przemyslaw Ustianowski, Damian Malinowski, Michał Czerewaty, Krzysztof Safranow, Maciej Tarnowski, Violetta Dziedziejko, Andrzej Pawlik

**Affiliations:** 1 Department of Nursing, Pomeranian Medical University, 70-111 Szczecin, Poland; 2 Department of Experimental and Clinical Pharmacology, Pomeranian Medical University, 70-111 Szczecin, Poland; 3 Department of Physiology, Pomeranian Medical University, 70-111 Szczecin, Poland; 4 Department of Biochemistry and Medical Chemistry, Pomeranian Medical University, 70-111 Szczecin, Poland

**Keywords:** gestational diabetes, polymorphism, *COBLL1*, *IRS1*

## Abstract

Gestational diabetes mellitus (GDM) is carbohydrate intolerance in pregnant women leading to various complications. Currently, there is a search for factors predisposing to GDM. Among them are genetic polymorphisms of genes involved in insulin secretion as well as carbohydrate metabolism. Due to the similar pathogenesis of GDM to type 2 diabetes (T2DM), genetic polymorphisms associated with T2DM are considered. The aim of this study was to examine the associations between the *COBLL1* rs7607980 T > C and *IRS1* rs2943641 T > C gene polymorphisms and the risk of GDM as well as selected clinical parameters in women with GDM. Additionally, we examined the expression of these genes in the placenta of women with and without GDM in correlation with selected clinical parameters. This study included 328 pregnant women with normal glucose tolerance (NGT) and 251 pregnant women with GDM diagnosed on the basis of a 75 g oral glucose tolerance test (OGTT) at 24–28 weeks gestation. There were no statistically significant differences in the distribution of *IRS1* rs2943641 gene polymorphisms between women with GDM and pregnant women with NGT. In the GDM group, we observed a decreased frequency of *COBLL1* rs7607980 CC homozygous women (CC vs. TC+TT, *p* = 0.048); however, there was no statistically significant difference in the frequency of alleles between women with GDM and the control group. There were no statistically significant associations between *COBLL1* rs7607980 gene polymorphism and clinical parameters in women with GDM. In GDM women with the *IRS1* rs2943641 TT genotype, fasting glucose levels were significantly higher than in women with CC and TC genotypes. There was no statistically significant difference in the expression of *COBLL1* and *IRS1* genes in the placenta between women with GDM and healthy women. There were no statistically significant correlations between *COBLL1* gene expression in the placenta and clinical parameters. The expression of *IRS1* correlated significantly with an increase in BMI during pregnancy. The results of this study suggest that *COBLL1* rs7607980 and *IRS1* rs2943641 gene polymorphisms are not significant risk factors for GDM in our population. The *IRS1* TT genotype may be associated with higher fasting glucose levels in women with GDM. Expression of the *IRS1* gene in the placenta positively correlates with an increase in BMI during pregnancy in women with GDM.

## 1. Introduction

Gestational diabetes mellitus (GDM) is a disorder of carbohydrate tolerance that occurs in pregnant women [1]. Risk factors for GDM include both genetic and environmental factors such as a family history of diabetes, age, and obesity [2,3,4,5]. Women with GDM have an increased insulin requirement due to both decreased insulin secretion as a result of pancreatic beta-cell dysfunction and tissue insulin resistance. In GDM, chronic inflammation is also observed, which adversely affects the fetus. Increased expression of inflammatory mediators has also been found in the placentas of women with GDM, especially those with obesity [5,6]. Hyperglycemia is associated with a number of adverse complications for the mother and fetus. Studies suggest that the pathogenesis of GDM is similar to that of type 2 diabetes mellitus (T2DM) [3,5]. The significant prevalence of T2DM in women with a history of GDM suggests that there is a common genetic background. Association studies have identified a number of genes related to pancreatic beta cell development, function, survival, and inflammation that affect the risk of T2DM and GDM [7,8].

The *COBLL1* gene encodes cordon-bleu protein-like 1. The *COBLL1* locus is genetically associated with the development of metabolic diseases and some cancers [9]. The molecular functions of the *COBLL1* gene remain unclear. It has been shown that SNP rs7607980 in the *COBLL1* gene affects insulin resistance [9,10]. The *COBLL1* rs7607980 gene polymorphism causes a thymine to cytosine substitution, resulting in a change of asparagine to aspartic acid at position 939 in the amino acid sequence. Several studies have examined this polymorphism in patients with diabetes type 2, insulin resistance, and metabolic syndrome [11,12,13,14].

Insulin receptor substrate (IRS) plays an important role in insulin-stimulated signaling pathways. IRS1 has been shown to regulate insulin action in adipose tissue, muscle, and pancreatic beta cells [15]. The *IRS1* gene has been shown to play a pathogenic role in the development of diabetes. In the *IRS1* rs2943641 variant, a C > T polymorphism has been identified. This polymorphism is located 500 kb downstream of the *IRS1* gene and is associated with elevated fasting hyperinsulinaemia and impaired insulin sensitivity [16,17,18]. Previous studies indicated that this polymorphism may be a risk factor for T2DM [19].

It has been shown that women with GDM have changes in the expression of some genes in the placenta, which may affect the clinical and biochemical parameters [20,21,22,23,24]. 

In this study, we examined whether *COBLL1* and *IRS1* gene polymorphisms are the risk factors for GDM development or affect clinical parameters in women with GDM. In addition, we aimed to investigate whether there are differences in the expression of these genes in the placenta between women with GDM and those with normal glucose tolerance and whether the expression of these genes in the placenta correlates with clinical and biochemical parameters.

## 2. Materials and Methods

### 2.1. Participants

This case-control association study included 579 pregnant women: 251 women with GDM and 328 women with normal glucose tolerance (NGT) treated in the Department of Obstetrics and Gynaecology, Pomeranian Medical University, Szczecin, Poland. The diagnosis of GDM was based on a 75 g oral glucose tolerance test (OGTT) at 24–28 weeks gestation, according to the International Association of Diabetes and Pregnancy Study Groups (IADPSG) criteria [25]. 

The diagnosis criteria of GDM were as follows: fasting plasma glucose of 92 mg/dL (5.1 mmol/L), 1 h plasma glucose of 180 mg/dL (10.0 mmol/L) or 2 h plasma glucose of 153 mg/dL (8.5 mmol/L). 

Of the pregnant women with GDM, 78% were treated with diet alone throughout pregnancy, and the remaining 22% used diet and insulin until delivery. Insulin therapy was implemented if morning glycemia was greater than 95 mg% (5.6 mmol/L) (for three consecutive days despite an adequate diet or glycemia after one of the main meals was greater than 140 mg% (7.8 mmol/L). The dose of insulin was adjusted according to serum glucose values by taking a starting dose of 0.7 IU/kg body weight/24 h. The dose was adjusted daily according to blood glucose levels measured four times a day.

Exclusion criteria were: diabetes type 1 and type 2, acute or chronic complications of diabetes, autoimmune and inflammatory diseases, neoplasmatic diseases, and chronic infections. All pregnancies were achieved by natural conception. 

In women included in the study, we analyzed clinical parameters such as fasting glucose, daily insulin requirement, body mass before pregnancy, body mass at birth, body mass increase during pregnancy, BMI before pregnancy, BMI at birth, BMI increase during pregnancy, newborn body mass, and APGAR score.

The subjects were educated about this study. Written informed consent was obtained from all subjects. The study was approved by the Ethics Committee of Pomeranian Medical University, Szczecin, Poland (KB-0012/40/14).

### 2.2. Determination of COBLL1 rs7607980 and IRS1 Gene rs2943641 Polymorphisms

Genomic DNA was extracted from 200 µL of whole blood samples using GeneMATRIX Quick Blood DNA Purification Kit (EURx, Gdansk, Poland). Genotyping was performed using allelic discrimination assays with TaqMan^®^ probes (Applied Biosystems, Waltham, MA, USA) on a 7500 Fast Real-Time PCR Detection System (Applied Biosystems, Waltham, MA, USA). In order to discriminate the polymorphisms, we employed TaqMan^®^ Pre-Designed SNP Genotyping Assays, including appropriate primers and fluorescently labeled (FAM and VIC) MGB™ probes to detect the alleles. All reactions were run in a final volume of 12 μL. To discriminate *COBLL1* rs7607980 and *IRS1* rs2943641 gene polymorphisms, TaqMan^®^ Pre-Designed SNP Genotyping Assays were used (assay IDs: C__29330585_10 and C___1533178_10).

Due to technical reasons, *IRS1* gene rs2943641 polymorphism was determined in 311 pregnant women with normal glucose tolerance and 231 with GDM.

### 2.3. Determination of COBLL1 and IRS1 Gene Expression in Placenta

#### 2.3.1. RNA Isolation

For this study, placentas were obtained from randomly selected 54 women (27 healthy women and 27 with GDM) who had a natural delivery after 37 weeks of gestation. All samples were collected at the Department of Obstetrics and Gynaecology, Pomeranian Medical University in Szczecin. After delivery, the whole placenta was placed in 0.9% NaCl and immediately transported to the Department of Physiology. The placental samples of approximately 100 mg were then excised from the maternal side of the cotyledons for RNA extraction. No visible connective tissue, vessels, and calcium deposits were detected. Total RNA was extracted from homogenates using the RNeasy Fibrous Tissue Mini Kit (Qiagen, Hilden, Germany) in accordance with the manufacturer’s protocol. 

The concentration and purity of RNA samples was determined by measuring the absorbance using a spectrophotometer Perkin Elmer Lambda Bio+ (PerkinElmer, Waltham, MA, USA).

#### 2.3.2. Real-Time Quantitative Reverse-Transcription PCR (RQ-PCR)

Isolated mRNA measuring 0.4 μg from each sample was reverse transcribed into cDNA in a total volume of 20 μL using a cDNA synthesis kit (RevertAid RT Kit, Thermo Scientific, Waltham, MA, USA) according to the manufacturer’s protocol.

The analysis of the quantitative expression of *COBLL1* and *IRS1* genes, as well as the reference gene, was performed using real-time RT-PCR on an ABI PRISM^®^ Fast 7500 Sequence Detection System (Applied Biosystems, Waltham, MA, USA) as previously described [21]. To normalize mRNA levels between different samples, we used β-2 microglobulin (*BMG*) as a reference gene. The reference gene was determined based on the available literature [22,23,24]. Each sample was analyzed in two technical replications and mean cycle threshold (CT) values were used for further analysis. Each reaction (20 μL) contained 2 μL of cDNA dilution. To calculate the values, the 2^−ΔCt^ method was used. Primers used for gene expression analysis by qRT PCR were as follow: *BMG*-F 5′-AATGCGGCATCTTCAAACCT-3′, *BMG*-R 5′-TGACTTTGTCACAGCCCAAGA-3′, *IRS1*-F 5′-ACAAACGCTTCTTCGTACTGC-3′, *IRS1*-R 5′-AGTCAGCCCGCTTGTTGATG-3′, *COBLL1*-F 5′-AGACCATAGTGAGAGTGAGTCC-3′, *COBLL1*-R 5′-GCTCTGTTGACATCCATCGCATA-3′. 

CT values ranges for qPCR reaction were as follows: *COBLL* control: 18.96–28.54 (23.31 ± 2.42), *COBLL* GDM: 18.12–31.40 (22.73 ± 3.36), *IRS1* control: 24.04–29.83 (27.09 ± 1.36), *IRS1* GDM: 23.81–30.92 (26.93 ± 1.54).

### 2.4. Statistical Analysis

The concordance of the genotype distribution with Hardy–Weinberg equilibrium (HWE) was verified with the Fisher exact test. Genotype and allele distributions were compared between groups using the chi-square test or Fisher exact test for tables with numbers in cells < 5. An odds ratio (OR) with 95% confidence interval (95% CI) was used as a measure of association between genotypes or alleles and GDM. Since the Shapiro–Wilk test showed that distributions of most of the quantitative variables were significantly different from normal distribution, they were compared between the genotype groups using the non-parametric Kruskal–Wallis test (when 3 genotype groups were analyzed) followed by Mann–Whitney U test and median with interquartile range (IQR) was used for descriptive statistics. Spearman rank correlation coefficient (Rs) was used to measure the strength of associations between placental expression and various clinical parameters. General Linear Model (GLM) was used for multivariate analysis after logarithmic transformation of quantitative variables with distributions significantly different from normal. Standardized β coefficients with 95% CI were calculated as a measure of association. *p* < 0.05 was considered statistically significant. 

The study with 251 GDM and 328 NGT pregnant women had sufficient statistical power to detect with an 80% probability true effect size of allelic association with GDM corresponding to OR 0.59 or 1.55 for *COBLL1* rs7607980. For a slightly lower number of 231 GDM and 311 NGT pregnant women genotyped for *IRS1* rs2943641, the respective effect size corresponded to OR 0.69 or 1.42. The study of placental gene expression in subgroups of 27 GDM and 27 NGT pregnant women had sufficient statistical power to detect with an 80% probability the true effect size of the difference in the expression between the subgroups corresponding to 0.8 standard deviation. Analysis of correlation between placental gene expression and clinical parameters had 80% statistical power to detect associations with an effect size corresponding to Spearman rank correlation coefficient ± 0.51.

*p*-values presented in the paper were not corrected for multiple testing. If such correction was applied, no association would remain statistically significant due to the large number of clinical parameters analyzed. Therefore the study should be treated as an exploratory one, requiring confirmation from further studies.

## 3. Results

The clinical characteristics of pregnant women are shown in Table A1 and Table A2 (Appendix A). 

The distributions of the studied polymorphisms were in the HWE (*p* > 0.05) and are shown in Table 1. There were no statistically significant differences in the distribution of *IRS1* rs2943641 gene polymorphism between women with GDM and pregnant women with NGT. In the GDM group, we observed a decreased frequency of *COBLL1* rs7607980 CC homozygous patients (CC vs. TC+TT, *p* = 0.048); however, there was no statistically significant difference in the frequency of alleles between GDM and control groups (Table 1).

We also examined the associations between the studied gene polymorphisms and clinical parameters: fasting glucose, daily insulin requirement, body mass before pregnancy, body mass at birth, body mass increase during pregnancy, BMI before pregnancy, BMI at birth, BMI increase during pregnancy, newborn body mass and APGAR score in women with GDM (Table 2 and Table 3).

There were no statistically significant associations between *COBLL1* rs7607980 gene polymorphism and clinical parameters. In women with the *IRS1* rs2943641 TT genotype, fasting glucose levels were significantly higher than in women with CC and TC genotypes. General linear model (GLM) analysis with *IRS1* rs2943641 TT genotype (compared to combined CC+CT genotypes) and log-transformed BMI before pregnancy as independent variables and log-transformed fasting glucose during pregnancy as dependent variable showed that both *IRS1* rs2943641 TT genotype (β = +0.20, 95% CI = 0.08–0.33, *p* = 0.0016) and BMI before pregnancy (β = +0.16, 95% CI = 0.03–0.28, *p* = 0.016) were independent factors positively associated with fasting glucose.

Additionally, we examined the expression of *COBLL1* and *IRS1* genes in the placenta of women with and without GDM. Expression of the *COBLL1* gene in women with and without GDM was: Median (IQR), 0.222 (0.104–0.595) and 0.154 (0.070–0.272), respectively. There was no statistically significant difference in the expression of *COBLL1* genes in the placenta between women with GDM and healthy women (*p* = 0.12). Expression of the *IRS1* gene in women with and without GDM was: median (IQR), 0.010 (0.005–0.025) and 0.009 (0.003–0.033), respectively. These differences were not statistically significant (*p* = 0.68).

We also examined correlations between expression in the placenta of *COBLL1* and *IRS1* genes in women with GDM and clinical parameters. There were no statistically significant correlations between *COBLL1* gene expression in the placenta and clinical parameters. Expression of the *IRS1* gene correlated significantly with an increase in BMI Table 4 and Table 5).

Furthermore, we investigated the association between placental *IRS1* and *COBLL1* gene expression and fetal sex. *COBLL1* gene expression was significantly higher in placentas of male fetuses; (median (IQR): 0.48 (0.15–0.81) vs. 0.18 (0.02–0.22), *p* = 0.02), whereas no association with *IRS1* gene expression was observed (median (IQR): 0.012 (0.004–0.17) vs. 0.006 (0.001–0.16), *p* = 0.15). Fetal sex was not associated with any of the studied clinical parameters.

## 4. Discussion

In this study, we examined associations between *COBLL1* rs7607980 and *IRS1* rs2943641 gene polymorphisms and the risk of GDM as well as selected clinical parameters in women with GDM. We observed decreased frequency of *COBLL1* gene rs7607980 CC homozygotes in women with GDM; however, there was no statistically significant difference in the distribution of alleles between women with and without GDM. We also examined associations between the studied polymorphisms and clinical parameters in women with GDM. Women with the *IRS1* gene rs2943641 TT genotype had increased fasting glucose levels. We also compared the expression of these genes in the placenta in women with and without GDM. These differences were not statistically significant. Additionally, we evaluated the correlation between placental expression of *COBLL1* and *IRS1* genes and clinical parameters in women with GDM. The expression of *IRS1* correlated significantly with an increase in BMI during pregnancy.

GDM is a disorder of carbohydrate metabolism that occurs during pregnancy, leading to numerous maternal and fetal complications. Therefore, a number of factors are being investigated that may be helpful in predicting the occurrence of GDM and in its diagnosis. Among the factors under consideration are genetic polymorphisms associated with T2DM risk. These include polymorphisms in genes affecting carbohydrate metabolism, insulin secretion, insulin resistance, and inflammation.

Previous studies have shown that *COBLL1* and *IRS1* gene polymorphisms are associated with T2DM risk and the development of metabolic syndrome. *COBLL1* is a gene that has not been widely studied to date. Previous studies have shown that the *COBLL1* locus is genetically linked to carbohydrate–lipid metabolism, the development of metabolic diseases, and some cancers [9]. The precise molecular functions of the *COBLL1* gene remain unclear. It has been indicated that the *COBLL1* gene may be a risk factor for T2DM. This gene may also affect lipid and carbohydrate metabolism. The *COBLL1* gene has been shown to be associated with body fat percentage and obesity, with subsequent metabolic and cardiovascular complications [9]. The *COBLL1* gene may also be associated with insulin resistance in human preadipocytes and adipocytes [13], and involved in metabolic syndrome and inflammation [10]. 

To date, *COBLL1* gene polymorphisms have not been widely studied as a risk factor for GDM or as a factor influencing disease course. Arora et al. examined *COBLL1* gene rs7607980 polymorphism in women with GDM from India. As in our population, there was no statistically significant association between this polymorphism and GDM risk in North Indian women. *COBLL1* gene rs7607980 polymorphism was associated with insulin resistance (HOMA-IR) [26]. In our population, we found no statistically significant association between COBL gene polymorphisms and the clinical parameters studied.

An association between the *COBLL1* rs7607980 C allele, lower serum insulin levels, and lower insulin resistance in overweight and obese children has also been indicated [14]. Our results showed a potentially protective effect of the *COBLL1* rs7607980 CC genotype on GDM risk, but this was not confirmed by allele analysis.

*IRS1* gene polymorphisms have been examined as risk factors for T2DM; however, the results differ between the populations studied. It has also been shown that this polymorphism may affect some parameters of carbohydrate and lipid metabolism and be involved in insulin resistance and metabolic syndrome. Ericson et al. indicated that *IRS1* rs2943641 may affect carbohydrate and fat intakes in patients with T2DM in a sex-specific fashion [16]. A protective association between the rs2943641 T allele and T2DM is restricted to women with a low carbohydrate intake and to men with a low fat intake. This polymorphism may also be associated with insulin resistance and HDL plasma levels in patients with T2DM and coronary artery disease [27]. It has been indicated that the rs2943641 C allele was associated with increased insulin resistance and hyperinsulinaemia, as well as reduced levels of IRS1 protein [28]. This SNP may also affect postprandial hyperglycaemia [29]. Mahmutovic et al. demonstrated that the rs2943641 C allele is associated with lower fasting glucose levels and HbA1c in non-drug-treated T2DM [30]. Furthermore, in our study, women with the rs2943641 CC genotype had lower fasting glucose levels.

Some meta-analyses have investigated the link between *IRS1* rs2943641 and T2DM. The meta-analysis by Zheng et al. indicated that rs2943641 T-allele carriers have a lower risk of insulin resistance, T2DM and metabolic syndrome [18]. They suggested that *IRS1* rs2943641 variants are associated with insulin resistance and are modulated by diet, so they may have important functions in various metabolic disorders, and dietary factors may modify these correlations [18]. In the meta-analysis by Li et al., the association between rs2943641 and T2DM risk was significant in a codominant model in a Caucasian population [19]. Ohshige et al. indicated that *IRS1* rs2943641 may be a common locus for T2DM across different ethnicities [31]. In our population, we found no statistically significant association between *IRS1* rs2943641 gene polymorphisms and GDM risk.

To date, *IRS1* rs2943641 gene polymorphism has not been widely examined in women with GDM. Prasad et al. have shown that the *IRS1* rs2943641 C allele is associated with increased insulin resistance (HOMA-IR). Their study demonstrated the genetic susceptibility of women with a history of GDM to impaired insulin secretion and sensitivity, and consequently to the development of diabetes [32]. In our study, the *IRS1* TT genotype was associated with higher fasting glucose levels in women with GDM. In addition, expression of the *IRS1* gene in the placenta correlated positively with an increase in BMI during pregnancy in women with GDM.

The results of our study suggest that *COBLL1* rs7607980 and *IRS1* rs2943641 gene polymorphisms are not significant factors influencing the risk of GDM development in our population. We have only shown a decreased frequency of the *COBLL1* rs7607980 CC genotype in women with GDM. However, there was no difference in allele frequency between the groups with and without GDM. In addition, in women with the *IRS1* TT genotype, higher fasting glucose levels were observed. We also found no differences in the expression of these genes in the placenta of women with GDM and healthy controls. However, expression of the *IRS1* gene correlated positively with an increase in BMI during pregnancy in women with GDM.

Unfortunately, these polymorphisms have not yet been widely studied in other populations as risk factors for GDM, and we cannot compare the results of our study with other populations. Most studies to date have focused on type 2 diabetes. Although there are many similarities in the pathogenesis of GDM and type 2 diabetes, however, there are also differences. The effect of genetic polymorphisms on the risk of GDM depends on a number of environmental factors that increase the risk of the disease, which may differ between the populations studied. Therefore, the impact of the polymorphisms studied may show inter-population differences.

## 5. Conclusions

The results of this study suggest that *COBLL1* rs7607980 and *IRS1* rs2943641 gene polymorphisms are not significant risk factors for GDM in our population. The *IRS1* TT genotype may be associated with higher fasting glucose levels in women with GDM. Expression of the *IRS1* gene in the placenta correlates positively with an increase in BMI during pregnancy in women with GDM

## Figures and Tables

**Table 1:** Distribution of *COBLL1* rs7607980 and *IRS1* rs2943641 genotypes and alleles in women with GDM and the control group.

	Control Group		GDM		*p* Value		OR (95% CI)	*p* Value
	*n*	%	*n*	%				
***COBLL1* rs7607980** **genotype**								
TT	246	75.00%	196	78.09%	0.11 *	CC+TC vs. TT	0.84 (0.57–1.24)	0.39 ^
TC	71	21.65%	53	21.12%		CC vs. TC+TT	0.23 (0.05–1.05)	0.048 *
CC	11	3.35%	2	0.80%				
**Allele**								
T	563	85.82%	445	88.65%		C vs. T	0.78 (0.55–1.10)	0.16 ^
C	93	14.18%	57	11.35%				
***IRS1* rs2943641** **genotype**								
CC	124	39.87%	86	37.23%	0.81 ^	TT+TC vs. CC	1.12 (0.79–1.59)	0.53 ^
TC	142	45.66%	109	47.19%		TT vs. TC+CC	1.09 (0.68–1.76)	0.72 ^
TT	45	14.47%	36	15.58%				
**Allele**								
C	390	62.70%	281	60.82%		T vs. C	1.08 (0.85–1.39)	0.53 ^
T	232	37.30%	181	39.18%				

^ χ2 test. * Fisher exact test. HWE: control group *p* = 0.065, GDM group *p* = 0.75 for *COBLL1* rs7607980, Fisher exact test. HWE: control group *p* = 0.72, GDM group *p* = 0.89 for *IRS1* rs2943641, Fisher exact test.

**Table 2:** Clinical parameters of women with GDM stratified according to *COBLL1* rs7607980 genotype.

Parameters	*COBLL1* rs7607980 Genotype		
	TT *n* = 196	TC + CC *n* = 55	TT vs. TC + CC
	Median (IQR)	Median (IQR)	*p* ^&^
Fasting Glucose [mmol/L]	5.11 (4.71–5.42)	5.14 (4.78–5.33)	0.79
Daily Insulin Requirement [IU/kg]	0.12 (0.0–0.34)	0.15 (0.0–0.38)	0.72
Body Mass before Pregnancy [kg]	70.0 (62.0–82.0)	67.0 (58.0–90.0)	0.92
Body Mass at Birth [kg]	84.0 (72.3–94.5)	82.0 (73.6–94.7)	0.99
Body Mass Increase during Pregnancy [kg]	11.7 (7.3–16.0)	11.0 (5.0–16.0)	0.39
BMI before Pregnancy [kg/m^2^]	25.5 (21.9–29.5)	24.6 (21.6–33.1)	0.99
BMI at Birth [kg/m^2^]	29.7 (26.5–34.4)	29.2 (26.5–34.9)	0.98
BMI Increase during Pregnancy [kg/m^2^]	4.1 (2.7–5.8)	3.8 (1.8–6.0)	0.42
Newborn Body Mass [g]	3320 (3050–3600)	3340 (2810–3630)	0.40
APGAR [0–10]	10.0 (9.0–10.0)	9.0 (8.0–10.0)	0.076

^&^ Mann–Whitney U test. IQR—interquartile range.

**Table 3:** Clinical parameters of women with GDM stratified according to *IRS1* rs2943641 genotype.

Parameters	*IRS1* rs2943641 Genotype						
	CC *n* = 86	TC *n* = 109	TT *n* = 36		CC vs. TC	CC vs. TT	TC vs. TT
	Median (IQR)	Median (IQR)	Median (IQR)	*p* ^#^	*p* ^&^		
Fasting Glucose [mmol/L]	5.12 (4.72–5.44)	5.09 (4.61–5.31)	5.26 (5.09–5.56)	0.022	0.43	0.041	0.0055
Daily Insulin Requirement [IU/kg]	0.11 (0.0–0.31)	0.13 (0.0–0.38)	0.08 (0.0–0.41)	0.97	0.82	0.85	0.95
Body Mass before Pregnancy [kg]	74.0 (65.0–84.0)	67.0 (59.0–77.0)	67.0 (60.0–87.0)	0.042	0.0097	0.34	0.54
Body Mass at Birth [kg]	88.0 (78.0–96.0)	78.0 (70.0–93.5)	83.5 (74.0–94.0)	0.029	0.0079	0.44	0.25
Body Mass Increase during Pregnancy [kg]	11.3 (7.0–15.6)	11.0 (7.5–15.0)	13.1 (5.2–17.5)	0.76	0.86	0.46	0.53
BMI before Pregnancy [kg/m^2^]	26.2 (23.8–31.0)	24.5 (21.5–28.0)	25.7 (21.5–33.1)	0.046	0.012	0.72	0.27
BMI at Birth [kg/m^2^]	31.6 (27.8–34.9)	28.4 (26.0–33.6)	31.2 (26.4–36.0)	0.026	0.011	0.98	0.081
BMI Increase during Pregnancy [kg/m^2^]	4.4 (2.4–5.9)	4.0 (2.7–5.7)	4.9 (2.1–6.6)	0.65	0.84	0.37	0.42
Newborn Body Mass [g]	3365 (2910–3640)	3250 (3020–3610)	3380 (3035–3585)	0.70	0.48	0.87	0.51
APGAR [0–10]	10.0 (9.0–10.0)	10.0 (9.0–10.0)	10.0 (9.0–10.0)	0.84	0.85	0.68	0.55

**^&^** Mann–Whitney U test. **^#^** Kruskal–Wallis test. IQR—interquartile range.

**Table 4:** Correlations between *COBLL1* expression in the placenta and clinical parameters in the GDM group (*n* = 27).

Parameters Correlated with Placental Expression of *COBLL1*	Rs	*p*
Age [years]	0.33	0.092
Fasting Glucose [mmol/L]	0.29	0.14
Daily Insulin Requirement [IU/kg]	0.05	0.81
Body Mass before Pregnancy [kg]	0.19	0.33
Body Mass at Birth [kg]	0.10	0.62
Body Mass Increase during Pregnancy [kg]	−0.01	0.98
BMI before Pregnancy [kg/m^2^]	0.13	0.53
BMI at Birth [kg/m^2^]	0.09	0.66
BMI Increase during Pregnancy [kg/m^2^]	−0.03	0.86
Newborn Body Mass [g]	0.00	1.00
APGAR [0–10]	0.10	0.63

R_s_—Spearman rank correlation coefficient.

**Table 5:** Correlations between *IRS1* expression in the placenta and clinical parameters in the GDM group (*n* = 27).

Parameters Correlated with Placental Expression of *IRS1*	Rs	*p*
Age [years]	−0.15	0.47
Fasting Glucose [mg/dL]	−0.01	0.98
Daily Insulin Requirement [IU/kg]	−0.19	0.34
Body Mass before Pregnancy [kg]	−0.13	0.51
Body Mass at Birth [kg]	0.06	0.75
Body Mass Increase during Pregnancy [kg]	0.45	0.018
BMI before Pregnancy [kg/m^2^]	−0.15	0.46
BMI at Birth [kg/m^2^]	0.01	0.97
BMI Increase during Pregnancy [kg/m^2^]	0.45	0.018
Newborn Body Mass [g]	0.23	0.26
APGAR [0–10]	−0.11	0.57

R_S_—Spearman rank correlation coefficient.

## Data Availability

Not applicable.

## Appendix A

**Table A1:** Clinical characteristics of women included in the analysis of *COBLL1* and *IRS1* gene polymorphisms.

Parameters	Control Group	GDM	*p* ^&^
	Median (IQR)	Median (IQR)	
Age [years]	30.0 (27.0–34.0)	32.0 (28.0–36.0)	0.00014
Height [cm]	166.0 (162.0–170.0)	165.0 (162.0–170.0)	0.75
Pregnancy Number	2.0 (1.0–2.0)	2.0 (1.0–3.0)	0.27
Fasting Glucose [mmol/L]	4.53 (4.29–4.78)	5.11 (4.76–5.41)	<0.0001
Daily Insulin Requirement [IU/kg]	0.0 (0.0–0.0)	0.11 (0.0–0.34)	<0.0001
Body Mass before Pregnancy [kg]	62.0 (55.0–70.0)	70.0 (60.8–83.0)	<0.0001
Body Mass at Birth [kg]	76.0 (69.0–85.5)	83.0 (73.0–94.8)	<0.0001
Body Mass Increase during Pregnancy [kg]	14.0 (11.0–17.0)	11.5 (7.0–16.0)	<0.0001
BMI before Pregnancy [kg/m^2^]	22.2 (20.4–25.2)	25.4 (21.9–30.2)	<0.0001
BMI at Birth [kg/m^2^]	27.6 (25.4–30.5)	29.7 (26.5–34.5)	<0.0001
BMI Increase during Pregnancy [kg/m^2^]	5.0 (3.9–6.2)	4.1 (2.5–5.9)	<0.0001
Newborn Body Mass [g]	3310 (3020–3630)	3320 (2990–3610)	0.97
APGAR [0–10]	10.0 (9.0–10.0)	10.0 (9.0–10.0)	0.13

**^&^** Mann–Whitney U test. IQR—interquartile range.

**Table A2:** Clinical characteristics of women included in the analysis of placental expression of *COBLL1* and *IRS1* genes.

Parameters	Control Group	GDM	*p* ^&^
	Median (IQR)	Median (IQR)	
Age [years]	30.0 (28.0–33.0)	31.0 (30.0–36.0)	0.12
Height [cm]	168.0 (162.0–170.0)	165.0 (160.0–170.0)	0.34
Pregnancy Number	2.0 (1.0–3.0)	2.0 (1.0–2.0)	0.92
Fasting Glucose [mmol/L]	4.39 (4.274.62)	5.17 (4.78–5.33)	<0.0001
Daily Insulin Requirement [IU/kg]	0.0 (0.0–0.0)	0.11 (0.0–0.31)	<0.0001
Body Mass before Pregnancy [kg]	65.0 (57.5–78.0)	72.0 (57.0–77.0)	0.59
Body Mass at Birth [kg]	79.0 (71.3–89.0)	84.0 (70.5–94.2)	0.48
Body Mass Increase during Pregnancy [kg]	12.0 (10.1–16.0)	12.0 (9.0–15.0)	0.95
BMI before Pregnancy [kg/m^2^]	23.9 (20.4–29.4)	26.0 (20.8–28.2)	0.35
BMI at Birth [kg/m^2^]	28.8 (26.2–31.5)	30.0 (26.2–34.5)	0.40
BMI Increase during Pregnancy [kg/m^2^]	4.3 (3.4–5.9)	4.4 (3.4–5.3)	0.65
Newborn Body Mass [g]	3300 (3120–3600)	3270 (3000–3680)	0.88
APGAR [0–10]	10.0 (9.0–10.0)	10.0 (9.0–10.0)	0.44
COBLL1 Expression in the Placenta	0.154 (0.070–0.272)	0.222 (0.104–0.595)	0.12
IRS1 Expression in the Placenta	0.009 (0.003–0.033)	0.010 (0.005–0.025)	0.68

**^&^** Mann–Whitney U test. IQR—interquartile range.
